# Monthly Report of Deaths in the City of Buffalo

**Published:** 1867-05

**Authors:** Sandford Eastman

**Affiliations:** Health Physician


### Monthly Report of Deaths in the City of Buffalo,

*For the Month of April,* 1867.

Whole number of deaths from disease, 125.

In addition to the above, 2 still-born were reported in the city.

SEX.—Males 78, Females 49. Causes of death.—Accident, by drowning, 1, Apoplexy, Cerebral 2, Bronchitis 3, Cancer 2, Cancer of the mouth 1, Cancer of the Stomach 1, Cancer of the Womb 1, Cholera Infantum 1, Cholera Morbus 1, Consumption 23, Convulsions 10, Croup 2, Debility 6, Disease of the Heart 3, Disease of the Lungs 1, Disease of the Stomach 1, Diphtheria 2, Dropsy, General 2, Dropsy of the Brain 1, Erysipelas 3, Fever, Puerperal 2, Fever, Typhoid 4, Fever, Typhus 1, Fracture of Skull 1, Gangrene of Lung 1, Hæmorrhage 1, Hæmorrhage from Lungs 2, Inflammation of Brain and Meninges 4, Inflammation of the Lungs 14, Inflammation of Lungs, Typhoid 4, Inflammation of Lungs and Pleura 1, Inflammation of Womb 1, Influenza 1, Infanticide 1, Kidneys, Bright's disease of, 1, Meningitis, Cere-Spin. 1, Measles 1, Old Age 8, Paralysis 3, Premature Birth 2, Rheumatism 1, Suffocation 1, Unknown 2. Total deaths from Diseases, 125. Still-Born, 2.

The following shows the number of deaths in the city in each month of the present year; the number in 1866, and the average of each month for five years, 1862 to 1866, inclusive:

| | 1867. | 1866. | 5 y's average. |
|---|---|---|---|
| January, - - - - - | 95 | 127 | 127 |
| February, - - - - | 130 | 132 | 125 |
| March, - - - - - | 155 | 100 | 120 |
| April, - - - - - | 127 | 123 | 128 |

The number of deaths in the first four months of the present year is 25 more than in the corresponding period of last year, and 7 more than the average for five years.

SANDFORD EASTMAN, Health Physician.

